# Comparison of prothrombin time (INR) results and main characteristics of patients on warfarin treatment in primary health care centers and anticoagulation clinics

**DOI:** 10.1186/1472-6963-13-85

**Published:** 2013-03-07

**Authors:** Kerstin Arbring, Srinivas Uppugunduri, Tomas L Lindahl

**Affiliations:** 1 Department of Medical and Health Sciences, Linköping University, Department of Acute Internal Medicine, County Council of Östergötland, Linköping, S581 85, Sweden; 2 Department of Clinical and Experimental Medicine, Linköping University, Department of Clinical Chemistry, County Council of Östergötland, Linköping, Sweden; 3 Department of Clinical and Experimental Medicine, Linköping University, Linköping, Sweden

**Keywords:** Oral anticoagulants, Treatment, Quality control, Warfarin

## Abstract

**Background:**

Oral anticoagulant therapy is used to prevent thrombosis in patients with atrial fibrillation (AF), venous thrombosis and prosthetic heart valves. The introduction of new therapies emphasizes the need to discern the best practice for the patients remaining on warfarin treatment. This study compares patient characteristics and therapeutic control in two settings managing warfarin treatment: Swedish primary health care centers (PHCC) and specialized anticoagulation clinics (ACC).

**Methods:**

Prothrombin time (PT) test results reported as International Normalized Ratio (INR) were collected for five consecutive days from patients on warfarin treatment; 564 PHCC and 927 ACC patients. Therapeutic control was calculated as PT test results in relation to intended therapeutic range (TR). Mann–Whitney Rank Sum Test and Chi^2^ test were used for statistical comparisons.

**Results:**

The PHCC patients were older than the ACC patients, 76 v. 70 years (p<0.01) with a predominance of men in both groups. The reasons for treating differed between the groups. Seventy-two percent of PHCC patients and 66% of ACC patients had a PT-INR within the intended TR (p<0.05). Men generally had better results than women (72% v. 63%, p<0.001) and particularly in the PHCC group v. the ACC group (78% v. 69%, p<0.01).

PT-INR above intended TR was significantly more common in the ACC setting, (p<0.05), for women overall (p<0.01), for women in the PHCC setting, and for ACC men (p<0.05).

**Conclusions:**

In this study both settings achieved good therapeutic control of warfarin treatment with a minor advantage for PHCC over ACC, and better results for men, especially in the PHCC setting. As patient characteristics differ between the PHCC and ACC, it is important to conduct further randomized studies to discern the best practice locally for warfarin management also after the introduction of new drugs.

## Background

Oral anticoagulant therapy (OAT) is used to prevent thrombosis in patients with atrial fibrillation (AF), venous thrombosis and prosthetic heart valves. The numbers of patients on OAT is steadily increasing worldwide. Although new drugs are being introduced, vitamin K antagonists (VKA) still predominate. The introduction of new therapies emphasizes the need to discern the best practice for patients remaining on warfarin treatment. In Sweden, the number of VKA-treated patients is approaching 200,000, almost 2% of the population, and the treatment is almost exclusively warfarin.

Good therapeutic control of VKA treatment, with regular prothrombin time (PT) tests reported as International Normalized Ratio (INR) within intended therapeutic range (TR) is imperative for minimizing adverse events (bleeding and/or thrombosis) [1-4].

A comprehensive study concluded that anticoagulation control in community-based patients with AF in the USA was similar to participants in clinical trials [5]. Prospective data on VKA management are limited, but most retrospective studies favor specialized anticoagulation clinics (ACCs) over “usual care” [1,4,6]. However, these results cannot be automatically extrapolated to Sweden, which has a high standard of VKA management and primary health care. This is exemplified by the recent Swedish national quality registry—Auricula—study, which showed that the quality of anticoagulation treatment in Swedish centers is high, with a mean TTR of 76.2% [7].

A previous Swedish study found no differences in bleeding complications between Swedish primary health care centers (PHCC) and ACC [8].

Although the general trend in Sweden is toward more centralized (ACC) management of VKA treatment, some counties prefer to use the patient’s PHCC instead. There is, theoretically, a third option, with patients testing themselves with or without self-management of dosing. However, this group is very small in Sweden, estimated at fewer than 1,000 patients.

Most study data about the therapeutic control of VKA treatment originates from centralized ACCs with computer registers for treatment data, leaving the more scattered and hard-to-access PHCC data relatively unexplored. At the time this study was conducted, both regimes, ACC and PHCC VKA management, coexisted in our region. Both settings offered similar services, including initiation/induction and continued treatment.

Delivery of care for patients requiring warfarin therapy can be organized according to completely different principles. The different settings should therefore be characterized and compared, preferably with actual “real-life” populations not subjected to interventional studies where the patient material is usually selected, before deciding which organization is best suited to satisfy local health care challenges. The introduction of new therapies, with an obvious impact on future management of anticoagulation therapy, further emphasizes the need to conduct studies to determine best practice for warfarin management.

The Rosendaal method [9], commonly used for Time in Therapeutic Range (TTR) calculations, is very cumbersome to perform in non-computerized settings. A cross-sectional method is more feasible in such a setting and was therefore chosen for this particular study.

The purpose of the present observational study was to perform a non-selective, snapshot cross-section comparison of the main patient characteristics and therapeutic control in the two different settings (PHCC and ACC) used for managing VKA treatment in a large population in our region. This also includes an investigation of potential differences between these two settings with respect to the proportion of PT-INR results within TR overall, and between men and women.

## Methods

We performed a cross-sectional study of an unselected material of patients on warfarin treatment managed by Swedish PHCCs and ACCs.

The study period was February 23 to February 27, 2004 (Monday to Friday, the days of the week when the ACCs and PHCCs are open). The raw material consisted of PT results from this week.

The treatment was monitored by regular analysis of PT using one combined thromboplastin from Medirox (Studsvik, Sweden). Instruments used were ACL 10 000 and ACL Futura from IL (Milan, Italy), Start from Diagnostica Stago (Asnières sur Seine, France) and Behnk Coagulator from Behnk Electronics (Norderstedt, Germany). Capillary blood samples were analyzed at the local laboratory or at the anticoagulation clinics; the quality was controlled by running control samples daily and by comparing split samples with the central laboratories at Linköping University Hospital or Jönköping Hospital.

All PT test results (reported as INR) from the central health care district of Östergötland County for the study period were collected from the laboratory information system (Flexlab™, TietoEnator, Linköping, Sweden); a total of 563 PHCC PT-INR test results. In case of multiple tests per patient, the first one was registered and subsequent test(s) were then removed. Forty-six duplicate tests were thus removed. The 16 PHCC centers in Östergötland each tested between 10 and 53 unique patients during the study period.

Information about the patients regarding intended TR and the reason for warfarin treatment was manually collected retrospectively from each PHCC. Eighteen test results from patients not on warfarin treatment, or where neither information regarding intended TR nor cause for treatment could be obtained, were further excluded.

All PT tests registered for the same period in the database of the ACC of Linköping University hospital and the ACC in Värnamo were collected (n=551 and 425, respectively) and duplicates were excluded as described above (n=81 and 26, respectively).

In addition, all PT tests for the same period and for warfarin-treated patients in the Eksjö area (one PHCC and one ACC) were reported to us. Duplicate tests were removed in Eksjö before the results were sent to us; only the PT-INR test results of unique patients (n=65 and 58 for the PHCC and ACC, respectively) were reported to us, along with anonymous patient data.

The reported cause for treatment for all patients was classified as venous thromboembolism (VTE, deep vein thrombosis and/or pulmonary embolism), atrial fibrillation (AF, with or without stroke), heart valve disease (Valve, including/primarily mechanical valve prosthesis) or other cause (Other, e.g. peripheral arterial embolism, other cardiac disease, stroke without any information on concomitant atrial fibrillation, or multiple causes).

All three ACCs and the PHCC in Eksjö recorded their patient data (including cause for treatment and intended TR) in a computer register, but the dosing was done manually in all centers. The remaining PHCCs used paper registers. No specific dosing algorithm was systematically used.

Statistics were calculated by Sigmaplot for Windows® 11.0 (Systat Software Inc.®) and Microsoft Office Excel® 2003 and 2007.

The study was approved by the Regional Ethical Review Board in Linköping.

## Results

The total number of PT-INR tests originating from unique patients (after exclusion as described in the Methods section) and the number of patients with known and reported TR and cause for treatment are shown in Table 1.

**Table 1:** Number of PT-INR tests (unique patients), known/reported therapeutic range and cause for treatment

**Location**	**PT-INR tests**	**Range stated**	**Cause stated**
PHCC Östergötland	499	480	495
PHCC Eksjö	65	65	65
**PHCC total** (men/women)	**564** (289/275)	**545** (282/263)	**560** (289/271)
ACC Östergötland	470	470	464
ACC Eksjö	58	58	58
ACC Värnamo	399	399	393
**ACC Total** (men/women)	**927** (540/387)	**927** (540/387)	**915** (531/384)

ACC = Anticoagulation clinics, PHCC = Primary health care centers.

The PHCC patients were older than the ACC patients, average age 76 and 70 years, median 78 and 74 years, respectively (p <0.001, Mann–Whitney Rank Sum Test). There were significantly more men in the PHCC group than in the ACC group, 51% and 58% respectively, (Chi^2^-test, p<0.01).

Reported causes for warfarin treatment for the PHCC and ACC groups are presented in Table 2. The causes differed between PHCC and ACC groups, with more heart valve disease patients in the ACC group.

**Table 2:** Causes for warfarin treatment for each setting

	**VTE**	**AF**	**Valve**	**Other**	**Total**
PHCC	162 (29%)	281 (50%)	17 (3%)	100 (18%)	560
ACC	222 (24%)	383 (42%)	157 (17%)	153 (17%)	915

VTE=Venous thromboembolism, AF=Atrial fibrillation, Valve=Heart valve disease, Other=Other or multiple cause for treatment.

The intended range was 2 (or 2.1) – 3 (or 3.1) for 93% (509/545) and 89% (826/927) in the PHCC and ACC groups, respectively, 2.5-3.5 for 1% (5/545) and 4% (41/927). Two and 11 patients were reported to have intervals with a width greater than 1.4 in the PHCC and ACC groups, respectively, and 19 and 23 patients were reported to have intervals with a width of 0.5 or less in the respective groups.

**Figure 1:**
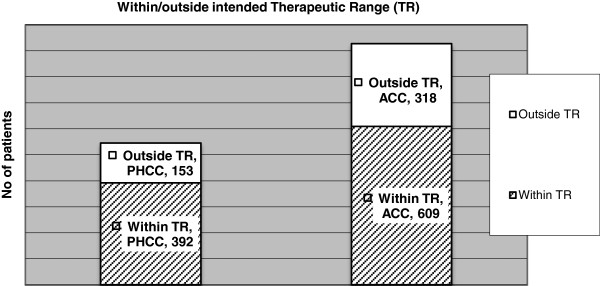
**PT-INR test results within and outside intended therapeutic range.** PHCC = Primary health care centers (PHCC) and ACC = Anticoagulation clinics (ACC settings).

When relating the PT-INR values to the intended TR, 72% of the PHCC patients and 66% ACC patients were within their TR, a small but statistically significant difference (Chi^2^-test, p<0.05), see Figure 1.

**Figure 2:**
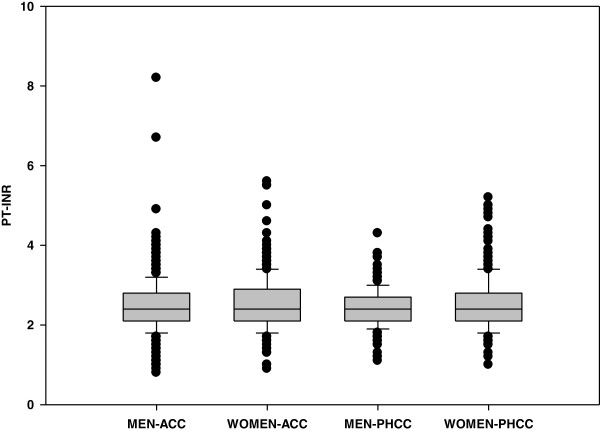
PT-INR test results for men and women in ACC and PHCC.

For the male patients 72% of the PT-INR values were within the intended TR, a significantly higher proportion compared with 63% for the female patients (Chi2-test, p<0,001). There was no significant difference between the PHCC and ACC groups of female patients, whereas the tests from the male patients were within intended TR at a significantly higher proportion in the PHCC group than in the ACC group: 78% in the PHCC group and 69% in the ACC group (Chi^2^-test, p<0.01). The distribution of crude PT-INR test results for these groups is shown in Figure 2, and the distribution of PT-INR test results related to the intended TR for the patient is shown in Table 3.

**Table 3:** Distribution of PT-INR test results for each setting

**Location**	**Below range**	**Within range**	**Above range**
**PHCC** (men/women)	**93** (42/51)	**392** (219/173)	**60** (21/39)
**ACC** (men/women)	**181** (100/81)	**609** (370/239)	**137** (70/67)

The number of PT-INR tests results above the intended TR was significantly higher in the ACC setting (15%) than in the PHCC setting (11%), (Chi^2^-test, p<0.05) and in women than in men overall (16% v. 11%, Chi^2^-test, p<0.01). No significant difference was found between settings for women, but for men the proportion of PT-INR test results above the intended range was significantly higher in the ACC setting than the PHCC setting (13% v. 7%, Chi^2^-test, p<0.05). In the ACC setting, there were no significant gender-related differences in test results. However, in the PHCC setting, the number of PT-INR tests above intended TR was significantly higher in women than in men (15% v. 7%, Chi^2^-test, p<0.01).

No significant difference was found related to PT-INR test results below intended TR either between the settings, between male and female patients overall, or in each respective setting.

The number of objectively high PT-INR results (PT-INR>5) was 1 and 5 (both less than 1%) for the respective PHCC and ACC groups, which was non-significant. Also no significant difference was found in the number of objectively low PT-INR results (PT-INR<1.8), 41 (8%) and 79 (9%) for the PHCC and ACC groups, respectively.

## Discussion

The latest American College of Chest Physicians (ACCP) Evidence-Based Clinical Practice Guidelines (9^th^ edition) [1,4] recognize that nonrandomized, retrospective studies and a few retrospective, comparative studies have reported better outcomes for the ACC setting compared with what is called “usual care” by personal physicians. They also found that four prospective, randomized controlled studies failed to show a significant difference in major hemorrhage or thromboembolism. The concluding ACCP Best Practices Statement [4] maintains that *“health-care providers who manage oral anticoagulation therapy should do so in a systematic and coordinated fashion, incorporating patient education, systematic INR testing, tracking, follow-up, and good patient communication of results and dosing decisions”* giving guidance on how it should be done rather than in which setting.

In this study of Swedish settings, we found a small advantage for the PHCC over the ACC and better results for men, especially in the PHCC setting. The reported characteristics in this study are different for the PHCC and ACC patients. We are aware that, since additional patient characteristics might differ between PHCC and ACC patients, lack of multivariate models that further explore associations between these potential differences and INR control is an obvious limitation in our study. Our aim was to study “real-life” VKA treatment patients as managed on a routine basis and unfortunately, additional patient characteristics (e.g. co-morbidities) were not registered systematically, neither at the ACC nor at the PHCC (and this is still the practice).

At the time of the study there was no systematic directing of more complicated patients to the ACC in our region. However, as our study was not randomized, we cannot exclude a referral bias. The older age of the PHCC patients may indicate a population with more concomitant disease, but we cannot exclude the possibility that the ACC patients may be medically equally complicated. The difference in characteristics may be of importance in itself; Poli [10] recently found better therapeutic control in AF than in VTE patients in an observational study on elderly patients.

Although both settings handle patients during the warfarin induction phase, with its potential out-of-range PT-INR values, we cannot exclude a slight overrepresentation of patients in the induction phase in the ACC compared with the PHCC. It can also be speculated that ACC patients might more often be subjected to different procedures that require a warfarin withdrawal followed by a “re-induction phase”. These temporary out-of-range PT tests would have less impact in a TIR interpolation model such as Rosendaal’s [9,11].

A high-standard PHCC setting offers many advantages as primary health care physicians may have a better opportunity to keep track of concomitant medication and other medical, practical and social circumstances that could have an impact on the warfarin treatment.

Male patients seem to do better, especially in the PHCC setting. We have no definite explanation for this. Small food portions with a higher risk of low vitamin K intake in women might contribute, but that does not explain a better outcome for men in the PHCC setting.

The data in this study was accumulated in 2004 and we cannot be sure if the results of our study are equally applicable to practice today. However, our study makes an objective comparison of two different settings at a time –point when both settings were equally experienced in managing warfarin treatment. Such a fair appraisal of both settings will help health-care professionals to choose the optimal local setting for future management of warfarin treated patients, alongside with the patients treated with the new oral anticoagulants.

## Conclusions

We conclude in the present study that both Swedish settings achieved good therapeutic control. Objective comparison of data from different settings, optimally head-to-head in a randomized, prospective study that eliminates differences in patient characteristics, is important before deciding which organization is best suited to satisfy local health care needs. This study and further studies comparing the different settings will assist health care professionals in choosing the optimal setting for future delivery of care for warfarin patients, based on local conditions.

The coming expected diversity of oral anticoagulants will present new organizational challenges for the delivery of adequate care for all patients requiring anticoagulation therapy. As of today, just above 2,000 patients in Sweden are being treated with dabigatran, the first new oral anticoagulant on the Swedish market. More drugs are being introduced. The advent of these new therapies will also pose difficult questions regarding optimal management of warfarin therapy for patients remaining on warfarin.

## Competing interests

The authors declare that they have no competing interests.

## Authors’ contributions

KA participated in the design of the study, collected and analyzed data and wrote the manuscript. TL participated in the design of the study, analysis of the data and writing of the manuscript. SU contributed to the analysis of data and writing of the manuscript. All authors read and approved the final manuscript.

## Pre-publication history

The pre-publication history for this paper can be accessed here:

http://www.biomedcentral.com/1472-6963/13/85/prepub
